# Amperometry methods for monitoring vesicular quantal size and regulation of exocytosis release

**DOI:** 10.1007/s00424-017-2069-9

**Published:** 2017-09-27

**Authors:** Hoda Fathali, Ann-Sofie Cans

**Affiliations:** Department of Chemistry and Chemical Engineering, Chalmers University of Technology, 42196 Gothenburg, Sweden

**Keywords:** Amperometry, Exocytosis, Fusion pore, Quantal size, Chromaffin cell, Artificial cells, Electrochemical cytometry, Intracellular electrochemical cytometry

## Abstract

Chemical signaling strength during intercellular communication can be regulated by secretory cells through controlling the amount of signaling molecules that are released from a secretory vesicle during the exocytosis process. In addition, the chemical signal can also be influenced by the amount of neurotransmitters that is accumulated and stored inside the secretory vesicle compartment. Here, we present the development of analytical methodologies and cell model systems that have been applied in neuroscience research for gaining better insights into the biophysics and the molecular mechanisms, which are involved in the regulatory aspects of the exocytosis machinery affecting the output signal of chemical transmission at neuronal and neuroendocrine cells.

## Introduction

Secretory vesicles are central in neuroscience and endocrinology. These organelles accommodate high concentrations of various neurotransmitter molecules and hormones serving as units of chemical message. Fusion of neurotransmitter-filled vesicles with the cell plasma membrane through a Ca^2+^-dependent process called exocytosis result in a rapid release of signaling molecules into the extracellular space and is a key process in intercellular communication [46]. To promote vesicle fusion of secretory cells in our bodies relies on a highly conserved molecular machinery based on the soluble *N*-ethylmaleimide-sensitive factor attachment protein receptors (SNARE) protein. These SNARE proteins consist of v-SNAREs localized in the vesicle membrane that associates with target t-SNAREs at the inner leaflet of the cell plasma membrane to initiate vesicle docking, fusion, and vesicle content release. These secreted signaling molecules diffuse from the release site of the emitting cell and bind to specific receptors at the surface of target cells inducing specific physiological reactions. In synaptic transmission, depending on type of neurotransmitters released, binding to target receptors at neighboring neurons may for instance serve to either excite or inhibit signal transmission in neuronal pathways. Hence, the secretory vesicle is an essential organelle that directly is influencing our brain function such as cognition, emotions, learning, and memory. Therefore, many neurological drugs are aimed to affect the quantity of neurotransmitters released and residing in the extracellular space by influencing the potency of neurochemical signals. The signal strength in cellular communication can be tuned at target cells receiving the signal via the sensitivity and level of the receptors expression in the cell membrane. Often, the amount of neurotransmitters released from an exocytosis event do not succeed to fully saturate the post-synaptic receptors in a synapse [38, 57, 93, 96]. Consequently, it has been realized that modulation of chemical signaling strength, thus can be regulated by the secreting cell through varying the amount of neurotransmitters released owing to the different modes of exocytosis that can be triggered [10, 14, 71, 72, 82, 93].

The maximal potency can be achieved from the classic view of exocytosis called “full exocytosis.” In this mode, the vesicle compartment upon fusion fully collapses into the plasma membrane releasing the entire vesicle content into the extracellular space [4, 11, 45, 66, 70, 77, 99]. In contrast to full vesicle content release, after vesicle fusion, the fusion pore can be controlled to stay open for a limited time and then close again. This allows a fraction of the vesicle neurotransmitter content to be released before the partly emptied vesicle is rapidly recycled while preserving its original shape [4, 78, 85]. The effectiveness of the chemical signal can thus be tuned by adjusting the pore size and the time it stays open [4, 14, 51, 60, 76]. The chemical signal can be minimized through the exocytosis mode “kiss and run,” where a transient 1–2 nm fusion pore that may also flicker, allows very small quantities of neurotransmitters to be secreted before the pore closes for vesicle reuse [3, 4, 12, 24, 34, 39, 66, 83, 91, 100]. A “moderate” signal can be delivered by means of a third mode of exocytosis where the transient fusion pore dilates into a larger pore extending the “kiss and run” in terms of fusion pore size and duration the pore is open, resulting in that more and yet not all of the vesicle content is expelled before the vesicle compartment is retrieved for recycling. This mode that more recently has been discovered and accepted has been termed many different names, e.g., “fuse-pinch-and-linger,” “extended kiss-and-run,” or “open and close exocytosis,” and seem to be the most prevalent mode of exocytosis used by for instance chromaffin cells [1, 11, 60, 61, 71, 78, 86, 89].

Depending on type of secretory cells and category of vesicles, quantification with carbon fiber microelectrode amperometry has estimated that about 30–60% of the vesicle content is released when this mode is triggered [51, 71, 72, 82]. Additionally, in chromaffin cells, fine-tuning of fusion pore size can serve as a molecular sieve, adding chemical selectivity of the compounds released by allowing molecules of a certain size to pass and others to be retained within the vesicle compartment [41, 69]. Hence, it is apparent that regulation of neurotransmitter release can directly be associated with mechanisms controlling the dynamics of the fusion pore affecting chemical signaling strength during intercellular communication and synaptic function. Therefore, to elucidate the regulatory mechanism and improve our understanding of what factors act to control fusion pore dynamics during exocytosis, it is important to identify the molecular drivers for vesicle-membrane interactions and the biophysics affecting that influences the exocytosis machinery. Many studies on secretion have, apart from neuronal cells also involved cell lines, simplified model systems and many major findings have been made through studies at chromaffin cells [15, 22, 53].

However, controversy still remains on the mechanisms for the regulatory aspect of exocytosis and the prevalence for different modes of exocytosis in secretory cells. This might relate to variance in the type of information provided by different analytical methods and limitations of instrumental techniques used in different experiments [69]. In addition, depending on the cell type and category of vesicles, the experimental conditions can vary widely. For instance, monitoring exocytosis release from small synaptic vesicles (40–60 nm in diameter) is a much greater challenge than from large dense-core vesicles (LDCVs) that vary in size from 80 to 300 nm in diameter [1, 82]. Therefore, in progress to clarify and verify results, development of new analytical tools improving sensitivity and spatiotemporal resolution for studying exocytosis is important. With individual methods varying in terms of different kinds of information achieved and technical limitations, an approach to improve the ability to overview the exocytosis process is to combine methodologies that provide complementary information. For instance, electrochemical methods offer quantitative and high temporally resolved detail information on fusion pore dynamics at the initial stages of exocytosis while reporting less on the later stages of exocytosis. In contrast, many imaging techniques have the capability to, with high spatial resolution, overview and track vesicle from docking, fusion, and vesicle content release and depending on the method used, also through vesicle recapture, but in comparison to electrochemical methods are limited in sensitivity and temporal resolution. Therefore, by merging electrochemical methods with imaging techniques offer the opportunity to simultaneously record the complementary information from both methods during secretion studies. For example, by combining high temporally resolved amperometry or electrophysiology, together with total internal reflection microscopy imaging, quantitative information of neurotransmitter release can be correlated with high spatial information on vesicle activity prior and post vesicle fusion, or by placing multiple lithographic electrodes at secreting cells electrochemical imaging capabilities of the exocytosis process can be created [42, 43, 49, 87, 92, 98, 101].

### Characterization of exocytosis with amperometry recording

**Fig. 1:**
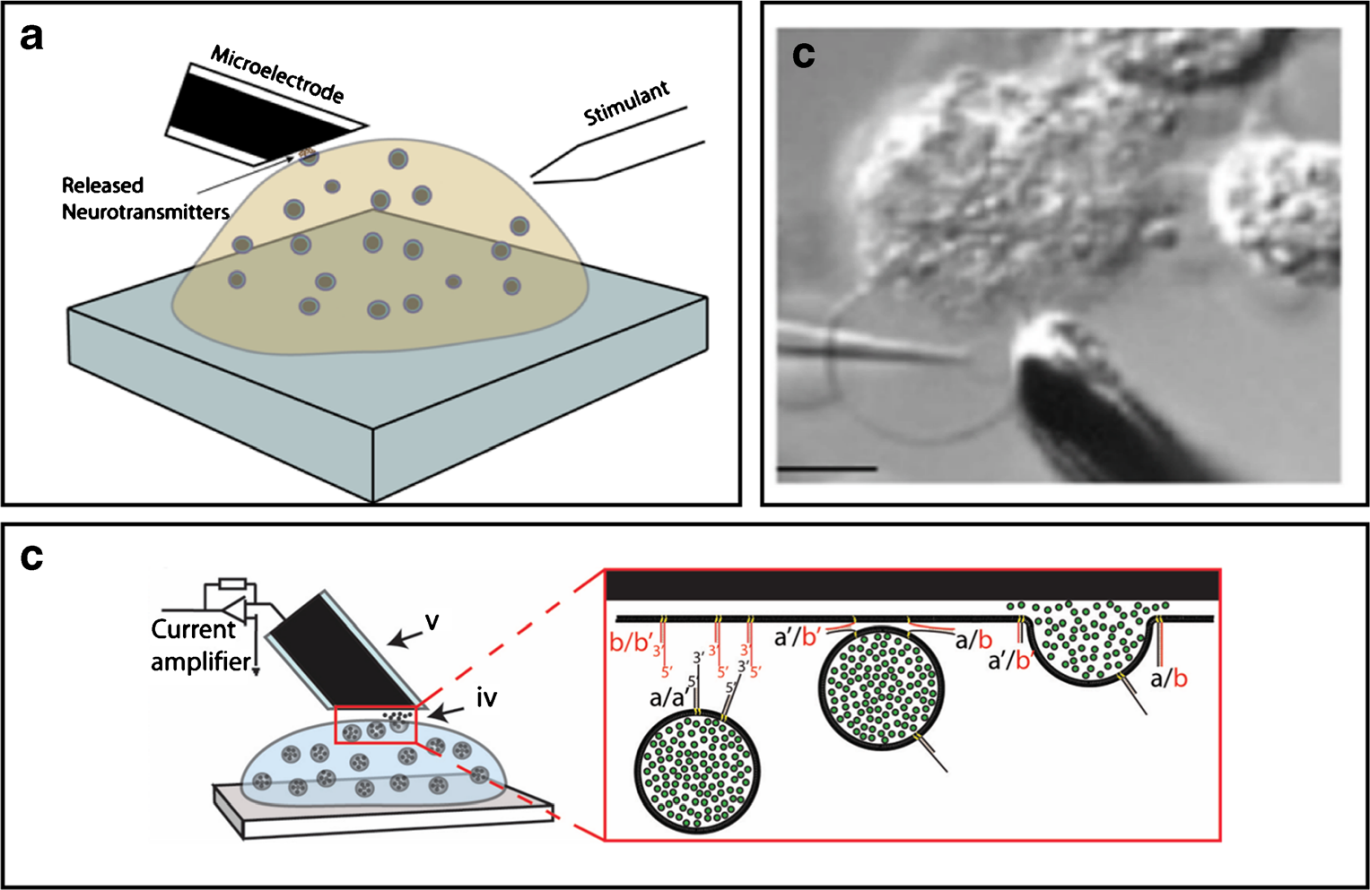
Here illustrates a few examples how the exocytosis process can be studied using live cells and synthetic cell models. **a** A schematic of an experimental set-up for amperometric measurement of exocytosis at live single cell [37]. **b** A DIC image of a cell model from bleb plasma membrane of a chromaffin cell, scale bar equals 10 *μ*m [60]. **c** A schematic of a protein-free cell model using DNA-zipper molecules mimicking the SNARE protein complex role in catalyzing vesicle fusion and neurotransmitter release [81]

Since the introduction of single-cell carbon fiber microelectrode recording of exocytosis activity at chromaffin cells by the Wightman lab [50, 90], amperometry has been a major analytical technique used in neurochemical research due to the simplicity of method implementation, the outstanding temporal resolution of sub-millisecond time scale and that this is the only existing method providing quantitative information of single-vesicle neurotransmitter release [63, 90]. In this electrochemical method, a carbon fiber microelectrode is placed in close contact to a secretory cell (as shown in Fig. 1a) and by applying an oxidation potential to the electrode, single vesicles release of signaling molecules that are electroactive, e.g., dopamine, adrenaline, noradrenaline, or serotonin, can be detected as individual current peaks at cells stimulated to exocytosis. By integrating the current versus time for each recorded amperometric peak, the total charge (*Q*) can be used to determine the number of neurotransmitter released from individual exocytosis events, by calculations using Faraday’s law expressed as *Q* = nNF, where *n* is the number of electrons transferred in the redox reaction (*n* = 2 for oxidation of catecholamines), *N* is the number of neurotransmitter molecules detected, and *F* is the Faraday constant (96,485 C mol^−1^). Typically, an amperometric current spike is marked by a rapid rise corresponding to a quick release of high-concentration neurotransmitter solution through the fusion pore as the pore dilates, followed by a slower decay representing the subsequent diffusion of molecules from the release site to the electrode surface [9, 63]. Analyzing the kinetic parameters from single current spikes present dynamic information on fusion pore formation, pore dilation and closing, which are important parameters for characterization of modes for exocytosis triggered at cells. Many amperometric peaks display a pre-spike feature called a “foot” signifying detection of neurotransmitter passing through an initial and stabilized narrow fusion pore, before the pore dilates releasing all or a fraction of the vesicle neurotransmitter content [5–7]. At partial vesicle release, a “post-spike foot” can sometimes be detected indicating that neurotransmitters are being released through a fusion pore that is closing for vesicle recapture and recycling [60]. As observed in both neuronal and chromaffin cells, a fusing vesicle can also be caught in a state of flickering that in amperometric recording can be displayed as a series of “stand-alone feet” signifying minuscule bursts of neurotransmitters release through a pore that by multiple rounds rapidly is opening and closing [63, 102]. The amplitude and lifetime of “foot features” portray information on fusion pore size, and kinetic information on fusion pore dynamics and stability. Hence, amperometry is a powerful analytical technique for quantitative single-cell exocytosis research and for characterization of mechanisms involved in regulation of neurochemical release.

### Amperometry to characterize the biophysics of the exocytosis process

With the key features of amperometry, this technique has been applied in many studies regarding the biophysics and key molecules involved in vesicle fusion and controlling fusion pore dynamics. For instance, investigations on the role of components associated to the sophisticated fusion machinery composed by SNARE protein promoting vesicle membrane fusion and questions regarding the involvement of SNARE protein in modulating neurotransmitter release, quantitative and kinetic measurements using amperometry has been central, with much of the work performed at chromaffin cells [13, 20, 36, 44, 67]. It is still debated what is the energy needed in terms of the minimum number of SNAREs for merging of a vesicle with the plasma membranes to initiate fusion and what is the potential role for SNARE proteins in deforming membranes into a high-curvature narrow fusion pore, as well as the possibility by the SNARE proteins to influence subsequent fusion pore dynamics. By introducing nanolipoprotein discs, a system with reconstituted v-SNARE proteins designed for complex binding to t-SNAREs expressed at the plasma membrane of chromaffin cells, a model system to study the cooperative effect of SNARE proteins forming and controlling fusion pore dynamics has been developed. This system has shown that very few SNAREs are needed to induce fusion pore formation and by the presence of a larger number of SNAREs can upon complex formation trigger fusion pore dilation [17, 25, 94, 95].

To gain a better understanding on what prevents the vesicle from fully collapsing with the plasma membrane when partial exocytosis is triggered, studies on the biophysics of fusion pore dynamics have facilitated to overview parameters that affect size, stability, and life time of the fusion pore [3, 26, 28, 29, 40]. It has been debated whether the fusion pore is lipidic or dominated by protein, with recent studies indicating it might be a mixture of both [16, 80]. Consequently, incubating cells with phospholipids of various geometrical shapes, e.g., cylindrical shapes, cone, or inverted cone shape, which modifies the spontaneous curvature of the cell membrane and directly affects membrane energetics and membrane dynamics of the highly curved fusion pore. Depending on lipid shape, whether facilitating positive or negative membrane curvature, alterations in membrane composition has shown to affect fusion pore size and act to slow or speed up the rate of fusion pore dilation during exocytosis [8, 23, 60, 76, 88]. Modifying the biophysics in terms of membrane rigidity for instance by adjusting the membrane cholesterol alters fusion pore dynamics, with lower membrane cholesterol concentrations accelerating exocytotic release and higher concentrations stiffening the membrane, stabilizes the fusion pore structure by increasing pore life time [18, 27, 84, 97]. Membrane tension is another key biophysical force that in neuroendocrine cells have shown to be a key regulatory force affecting the mechanism for driving fusion pore dilation and when applied by ATP-driven actin assembly forces at the plasma membrane reduces the secretory vesicle size during the exocytosis release process [21, 73]. The influence of biophysics on the exocytosis process has also been studied in controlled systems by a simplified cell model system created from protein-free giant liposomes (as presented in Fig. 1c) or cell plasma membrane “blebs” of chromaffin cells, where membrane is kept at higher complexity and more similar to cell membranes in comparison to protein-free cell models (as shown in Fig. 1b) [22, 59, 65, 80]. Blebs are plasma membrane vesicles that can be achieved from subjecting cells in culture to different kinds of stress, for instance by chemical treatment using formaldehyde, which disrupt cytoskeletal attachment points to the plasma membrane and thereby induces formation of micron-sized cell plasma membrane protrusions at the cell that can serve as cell model membranes [79].

In a protein-free cell model, systems showed that membrane rigidity highly influences the speed of exocytosis and that membrane dynamics alone can drive the later stages of exocytosis and influence the stability of the fusion pore before dilation [23, 65, 81]. In experiments using a bleb cell model demonstrated that a threshold in membrane tension can reversibly dictate if full or partial exocytosis was triggered [59], and by altering the membrane rigidity by changing the membrane cholesterol concentration, higher cholesterol levels significantly enhanced fusion pore stability and life time when partial exocytosis release was triggered [59, 65]. Although much of the work so far has been performed on gaining a better understanding of membrane biophysics of exocytosis, the biophysical properties of the secretory vesicle are still quite poor and therefore future studies with this focus might also serve to better explain many of the complex mechanisms involved in regulating neurotransmission.

### Secretory vesicle quantal size

The limiting amount of neurochemicals that can be released at an exocytosis event is naturally also directly related to the maximum number of neurotransmitter molecules that can be stored in a single vesicle compartment in the first place. This is often referred to as the vesicle “quantal size” referring to the basic unit of neurotransmitters that can be released as a discrete uniform “quanta” per exocytotic event [33, 48]. The secretory vesicles in neuroendocrine and neuronal cells are classified into two main groups; one is small synaptic vesicles containing low-molecular weight neurotransmitters such as dopamine, serotonin, norepinephrine, acetylcholine, or glutamate and mediate fast synaptic transmission. The other group is large dense-core vesicles (LDCVs) that typically contain monoamines (dopamine, adrenaline, noradrenaline, histamine, serotonin) and/or neuropeptides [2, 61]. Vesicles very efficiently accumulate high concentration of neurotransmitters, driven by proton and/or electrical gradients across the vesicle membrane depending on the type of neurotransmitter that is loaded into the vesicle [2, 32, 68]. The concentration of neurotransmitters in vesicles of chromaffin cells from adrenal glands is estimated to about 0.5–1 M [2, 32, 47, 62]. How the vesicle can perform this act and maintain charge neutrality and osmotic balance is still debated [19, 31, 35, 56]. The quantal size can be tuned using pharmacology enhancing or blocking the passage of neurotransmitters by specific transporter protein into the vesicle compartment or by affecting the proton/electrical gradient across the vesicle membrane and consequently affect the amount of transmitters released into the synaptic cleft [30, 51, 58, 74]. For neuroscience and pharmacological research, to elucidate how quantal size is maintained under physiological conditions and monitoring the effect on vesicle neurotransmitter content in response to potential drugs, analytical tools that are able to quantify the absolute neurotransmitter content in vesicles are crucial. However, universal methods for quantitative analysis of secretory vesicle content remain a challenge due to the variation in content and size of secretory vesicles. Quantitative measurement from the smallest of these organelles, the synaptic vesicle thus requires more sensitive and faster analysis techniques than probing the quantal content of larger dense-core vesicles in for instance neuroendocrine cells. Although fluorescence imaging with fluorescent dyes reporting on alteration in pH and membrane potential can offer real-time measurement of relative changes in vesicle quantal size [75], the only quantitative methods that count the absolute number of neurotransmitters encapsulated inside single secretory vesicles compartments are amperometry-based techniques [32, 51, 72]. In comparison to imaging methods for studying vesicle neurotransmitter loading, amperometry methods are to date limited to analysis of vesicles filled with neurotransmitters that are electroactive and performing single point-in-time measurements in contrast to measurements of relative dynamic changes in vesicle neurotransmitter content over time [32, 51, 72, 75].

### Amperometry techniques for quantification of vesicle quantal size

**Fig. 2:**
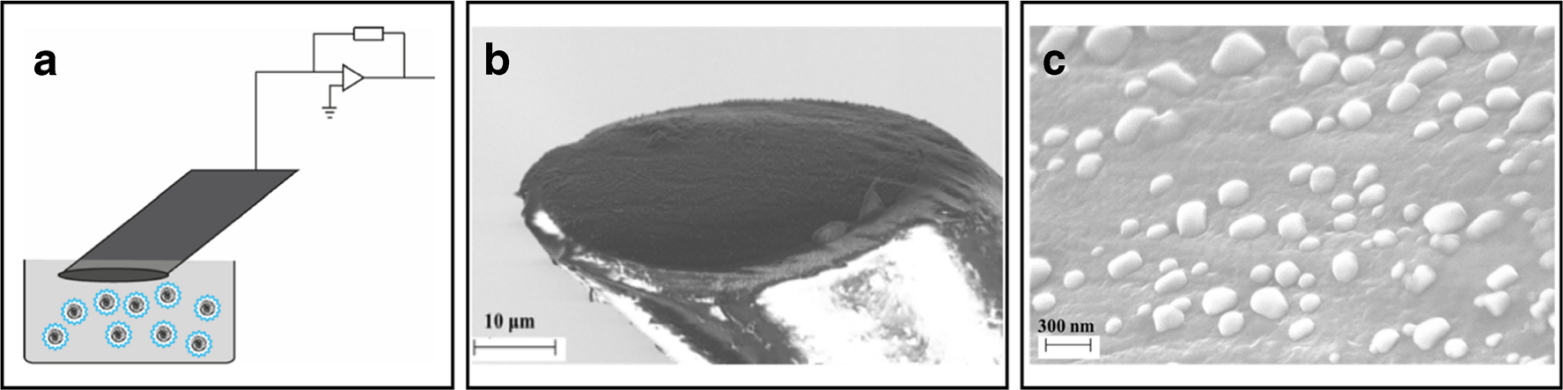
**a** A schematic of experimental set-up for electrochemical cytometry of isolated secretory vesicles. **b** Scanning electron microscopy (SEM) image of a 33-micrometer carbon fiber disc electrode after exposing to LDCVs sample from PC12 cells. **c** Magnified SEM image illustrating fixed isolated vesicles adsorbed to the surface of a carbon disc electrode

To present answers to the central question on what regulates secretory vesicle quantal size and for gaining a better understanding on how cells may tune their signals through triggering different modes of exocytosis during neurotransmission, methods that can provide both quantitative information on the absolute neurotransmitter content of vesicles and to be complemented with methods monitoring the amount neurotransmitters released through secretion are needed. Therefore, to complement amperometry measurements of exocytosis that count the number of neurotransmitters released during an exocytosis event, development of methods for monitoring the total vesicle neurotransmitter content has been key, and was first employed by combining cell-attached patch clamp capacitance measurement together with amperometry recording [4]. Here, quantification was achieved by correlating the amperometric current transients from neurotransmitter release with increases in capacitance steps as a result of single vesicle fusion with the cell plasma membrane. The quantitative analysis from these experiments revealed partial release as an alternative mode of exocytosis. Subsequently, this method was further developed by placing an amperometric carbon fiber microelectrode inside a patch pipette to provide dual detection of changes in membrane capacitance from single-vesicle exocytotic fusion events together with amperometric quantitative information on the number of neurotransmitters molecules secreted from each single vesicle into the patch pipette [2]. By comparing the vesicle membrane surface area from the patch clamp recording and correlating to the simultaneous amperometric recording of these measurements, the neurotransmitter concentration of single vesicles was calculated. The patch amperometry technique was further used to determine quantal release from different modes of exocytosis [3]. However, for quantal size analysis, it requires that the full vesicle content be secreted during exocytosis and this technique has a low throughput in the number of vesicle that can be analyzed per measurement. Thereafter, for vesicle content analysis, an amperometric method based on separation of isolated vesicles using capillary electrophoresis with amperometric detection [70–72]. This technique referred to as “electrochemical cytometry” might suggest by the name that this method involves chemical analysis of single cells, but is rather a method for analysis of subcellular structures and here aimed for neurotransmitter content analysis of secretory vesicles. In this method, a sample of isolated vesicles is injected into a quartz capillary and by applying an electric field, the vesicles migrate through the capillary and elute one-by-one onto a microfluidic platform. Here, individual vesicles subsequently are subjected to a lysis buffer, which induce vesicles to rupture, resulting in a current spike for each vesicle lysed that discharge the vesicle content of neurotransmitters at the surface of an amperometric carbon fiber electrode. From Faraday’s law, the total charge detected for each current spike is used to calculate the quantal size of each single vesicle. Using this method, one single sample of isolated vesicles injected onto the capillary provides quantitative measurement for a very large number of vesicles and therefore provide both very good throughput and statistical data for each measurement. Experiments using this technique, revealed that, by comparing the average quantal size of synaptic dopamine vesicles from striatum or from LDCVs in PC12 cells, and comparing to the amount neurotransmitters that is secreted at exocytosis, indicated that only a part of the vesicle content was released at these cells where previously full exocytosis was assumed to be the most prevalent mode of exocytosis [70, 71]. Hence, the electrochemical cytometry method in combination with amperometry recording of exocytosis at single cells offers a means for studying regulatory mechanisms by secretory cells and to give potential answers to the debate on the prevalence and what triggers the different modes of exocytosis at various cell types. The electrochemical cytometry method was recently further simplified by the discovery that placing an amperometric carbon fiber microelectrode in the solution of isolated large dense-core vesicles from chromaffin cells (shown in Fig. 2), vesicles collide, adsorb, and subsequently stochastically rupture and thereby releasing the vesicle content at the electrode surface for amperometric detection and quantification [32]. The frequency of vesicle rupture using this method is dependent on the applied potential at the carbon fiber electrode and is enhanced when the applied potential is increased from + 0.7 to + 0.9 V at the electrode surface [55]. Moreover, rupturability can also be amplified by excitation of augmented fluorophore-labeled lipids in the vesicle membrane or by modification of membrane permeability by incubation of vesicles with dimethyl sulfoxide [64]. Although by catalyzing the efficiency in vesicle rupture during measurements can alter current spike kinetics, by influencing the speed neurotransmitters are release from a vesicle, the total charge detected per vesicle is not altered and hence does not affect vesicle quantal size analysis. A mechanism for this methodology has been suggested that after vesicle adsorption to the electrode surface, the applied redox potential at the electrode surface induces electroporation of the vesicle membrane if the vesicle is in close enough to the electrode surface and that causes an irreversible pore dilation leading to vesicle rupture. Although the detection efficiency of this method has not yet experimentally been verified, the consistency in vesicle quantal size at various experimental conditions tested, e.g., applying various amperometric potentials or excitation of fluorophores in the vesicle membrane, in conjunction with the fact that a larger number of neurotransmitters are detected to be accumulated in the vesicle compartment compared to the amount neurotransmitters that is expelled at exocytosis, indicates that if not all, a major part of the vesicle neurotransmitter content is detected by this method [52, 55]. These new electrochemical cytometry tools provide the ability to quantify changes in quantal size of isolated vesicles at controlled experimental conditions and to study how vesicle responds to changes in chemical and physical environments as well as in response to pharmacological agents.

### Intracellular electrochemical cytometry

**Fig. 3:**
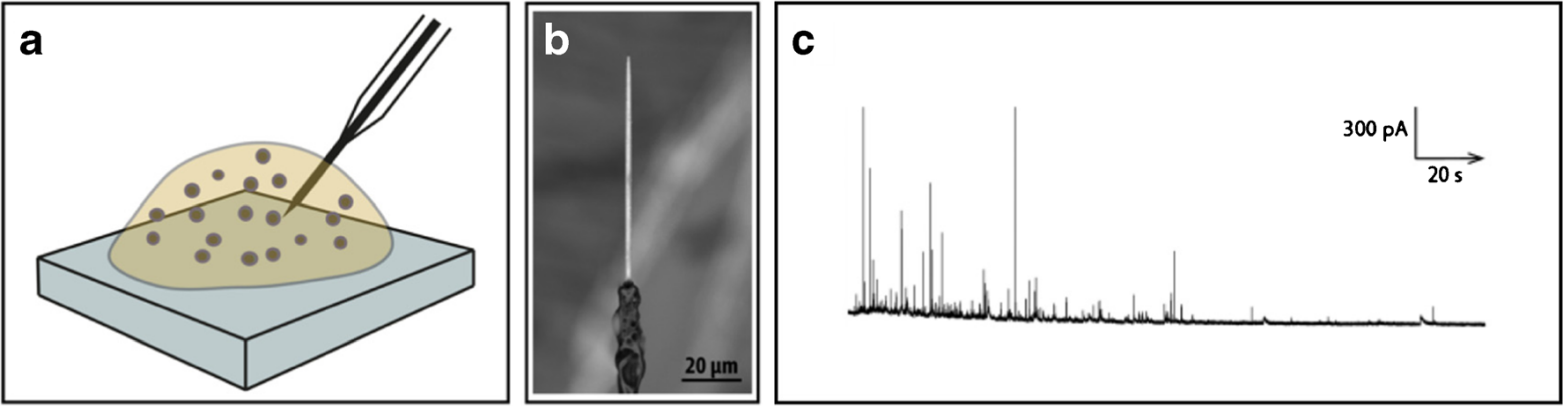
**a** A schematic of an intracellular electrochemical cytometry experiment showing placement of a carbon fiber electrode in the cytoplasm of a cell. **b** A SEM image of a nanotip conical carbon fiber microelectrode with a tip size of electrode about 100 nm and a tip length of 30–100 *μ*m. **c** A representative amperometric trace of intracellular cytometry recording from a chromaffin cell [37]

The secretory vesicle responds to transport and changes in gradients of analytes across the vesicle membrane, where components from the cytosol and the vesicle are shuttled by specific membrane transport proteins that are controlled and driven by proton or potential gradients across the vesicle membrane. Hence, the quantal size of an isolated vesicle might be affected when isolated into a defined isosmotic sample solution in comparison to when situated in the native environment of the cell cytoplasm, where the vesicles quickly can respond to a myriad of biochemical reactions in the surroundings. For instance, in the cytoplasm, a constant access of ATP feed the ATPase proton pump keeping a proton gradient across the vesicle membrane. From a constant synthesis, neurotransmitters are also actively pumped from the cytosol and into the vesicle compartment. To be able to study quantal size of vesicles in their native environment, the electrochemical cytometry method was further developed for in situ analysis of live single cells [51]. By flame etching a carbon fiber microelectrode into a tens of micrometers long sharp tip, with the tip size about 100 nm, this spear-shaped electrode was placed into the cytoplasm of live cells through applying a gentle mechanical force (as shown in Fig. 3). Similar to the electrochemical cytometry method, by applying an oxidation potential to the electrode surface, vesicles in the cytoplasm collide, adsorb, rupture, and release the vesicle content at the surface of the carbon electrode allowing quantification of vesicle neurotransmitter content in situ of live cells. In comparison to electrochemical cytometry of isolated vesicles, here, the sample matrix is very different to bulk conditions, as the electrode is placed in a highly crowded environment and the etched electrode provides a surface area with high curvature shape as opposed to using a disc microelectrode. Hence, it is important to keep in mind that electrode sensitivity differs between these two methods and therefore comparison with absolute measures is preferentially performed using the same type of electrode. For instance, an amperometric flame-etched carbon fiber nanotip electrode was placed at the cell surface of a secreting PC12 cells treated with *L*-3,4-dihydroxyphenylalanine (L-DOPA) compared to vesicle quantal size using the same electrode for intracellular electrochemical recording [51]. As L-DOPA is a precursor for dopamine production in cells, which consequently upon transformation into dopamine is selectively and rapidly transported by the vesicular monoamine transporter protein (VMAT) into the vesicle compartments for storage, treatment of cells with this drug increased vesicle quantal size and showed in these experiments that also an increased amount of neurotransmitters released was observed. These measurements also confirmed that only part of the vesicle content is released from PC12 cells at exocytosis and that the ratio of neurotransmitter released by L-DOPA-treated cells was similar to untreated cells. Therefore, this suggests that the exocytosis mode used by these cells is preserve during drug treatment [51]. Intracellular cytometry was also used to shed some light on the affect of osmotic pressure on secretory vesicles. Previously, it was observed that secretory cells respond to hypertonic stress by reducing exocytosis activity and the amount neurotransmitters released at exocytosis. Assumptions were made that alteration in membrane biophysical properties and cytosolic molecular crowding caused the reduction in the amount neurotransmitters released and in exocytosis activity, while assuming that secretory vesicles in the cytoplasm of cells do not sense or respond to extracellular osmotic stress. To provide better understanding regarding the effect of osmotic stress on vesicle quantal size at vesicles in their native environment, the total content of neurotransmitter in vesicles was probed directly in the cytoplasm of live cells before and after exposure to osmotic stress [37]. These quantitative measures of alteration in quantal size were compared to the amount neurotransmitters released during exocytosis at chromaffin cells and vesicle size measurements using transmission electron microscopy (TEM) imaging were all performed under the same different experimental conditions. As the result, the ability to compare relative changes in quantal size, both before and after exocytosis at different osmotic conditions, showed that vesicles respond to osmotic stress by shrinking and reducing the quantal size with the aim to maintain a constant vesicle neurotransmitter concentration and therefore less neurotransmitters are released by cells during osmotic stress [37]. Intracellular electrochemical cytometry was also used to complement nano scale secondary ion mass spectroscopy (NanoSIMS) that together with TEM imaging analysis was used to correlate chemical imaging of single vesicle granules and studying kinetics and mechanism for catecholamine loading into dense-core vesicles from chromaffin cells, where pharmacological treatment with isotope-labeled L-DOPA at shorter and longer times and together with the VMAT blocker reserpine was applied. Combining all three methods, an overview picture was created how catecholamine quickly loads into the halo solution of these vesicles from where a certain permeability for catecholamine exists over the vesicle membrane and that catecholamines with slower kinetics bind to the dense core protein that serve as a matrix to accumulate a neurotransmitters at high concentration in these vesicles while reducing the osmotic pressure inside the vesicle compartment [54].

## Conclusions

It has been realized that emitting cells have the ability to take part in regulation of signaling strength during intercellular communication both through regulation of content release by the mode of exocytosis triggered and by the amount of signaling molecule that is stored in secretory vesicles. In gaining a better overview in how this regulation is accomplished, analytical tools that can provide methods to study molecular mechanisms controlling fusion pore dynamics and factors influence loading and storage of neurotransmitter in secretory vesicle compartments are crucial. This is also important for better understanding of how drug treatment and disease might act and affect the regulatory aspects of neurochemical release, as well as characterizing the physiological effects in response to regulatory neurotransmission. In this quest, to study these dynamic processes in cells and secretory vesicles, cell model systems and analytical techniques that can provide sufficient sensitivity and temporal resolution to probe these ultra-small systems at physiological relevant conditions will be of great importance to clarify the essential regulatory aspects of exocytosis.
